# Production and characterization of specific monoclonal antibodies binding the *Plasmodium falciparum* diagnostic biomarker, histidine-rich protein 2

**DOI:** 10.1186/1475-2875-13-277

**Published:** 2014-07-18

**Authors:** Chiuan Herng Leow, Martina Jones, Qin Cheng, Stephen Mahler, James McCarthy

**Affiliations:** 1 QIMR Berghofer Medical Research Institute, Brisbane, Australia; 2 School of Medicine, University of Queensland, Brisbane, Australia; 3 Institute for Research in Molecular Medicine (INFORMM), Universiti Sains Malaysia, Penang, Malaysia; 4 Australian Institute for Bioengineering & Nanotechnology (AIBN), University of Queensland, Brisbane, Australia; 5 Drug Resistance and Diagnostics, Australian Army Malaria Institute, Brisbane, Australia

## Abstract

**Background:**

Early and accurate diagnosis of *Plasmodium falciparum* infection is important for providing appropriate treatment to patients with malaria. However, technical limitations of currently available diagnostic tests limit their use in control programs. One possible explanation for the vulnerability of current antibodies used in RDTs is their propensity to degrade at high ambient temperatures. Isolation of new antibodies with better thermal stability represents an appealing approach to improve the performance of RDTs.

**Methods:**

In this study, phage display technology was deployed to isolate novel binders by screening a human naïve scFv antibody library against recombinant *Plasmodium falciparum* histidine rich protein 2 (rPfHRP2). The isolated scFv clones were reformatted to whole IgG and the recombinant mAbs were produced in a mammalian CHO cell expression system. To verify the biological activity of these purified recombinant mAbs, range of functional assays were characterized.

**Results:**

Two unique clones (D2 and F9) were isolated after five rounds of biopanning. The reformatted and expressed antibodies demonstrated high binding specificity to malaria recombinant PfHRP2 and native proteins. When 5 μg/mL of mAbs applied, mAb C1-13 had the highest sensitivity, with an OD value of 1, the detection achieved 5 ng/mL of rPfHRP2, followed by mAbs D2 and F9 at 10 ng/mL and 100 ng/mL of rPfHRP2, respectively. Although the sensitivity of mAbs D2 and F9 was lower than the control, these recombinant human mAbs have shown better stability compared to mouse mAb C1-13 at various temperatures in DSC and blot assays. In view of epitope mapping, the predominant motif of rPfHRP2 recognized by mAb D2 was AHHAADAHHA, whereas mAb F9 was one amino acid shorter, resulting in AHHAADAHH. mAb F9 had the strongest binding affinity to rPfHRP2 protein, with a K_D_ value of 4.27 × 10^−11^ M, followed by control mAb C1-13 at 1.03 × 10^−10^ M and mAb D2 at 3.05 × 10^−10^ M.

**Conclusions:**

Overall, the performance of these mAbs showed comparability to currently available PfHRP2-specific mouse mAb C1-13. The stability of these novel binders indicate that they merit further work to evaluate their utility in the development of new generation point of care diagnosis of malaria.

## Background

Malaria, caused by five species of human *Plasmodium* and, of these species, infection with *Plasmodium falciparum* is the most prevalent and lethal, causing significant morbidity and mortality worldwide [1]. Therefore, it is crucial to understand the important parameters in the transmission of the disease, and develop effective diagnostic strategies for its prevention and control.

Today, rapid diagnostic tests (RDTs) are increasingly used for malaria diagnosis by detection of parasite biomarkers as they offer a result within 20 minutes. In these tests, *Plasmodium* lactate dehydrogenase (pLDH), and fructose 1,6-biphosphate aldolase (Aldolase) are commonly used as candidate targets for detection of infection with other *Plasmodium* species [2-4]. However, *P. falciparum* histidine-rich protein 2 (PfHRP2) is a biomarker that is predominantly used as a target for detection of *P. falciparum* infection [5,6]. PfHRP2 is a water-soluble protein that is produced by the asexual stages and gametocytes of *P. falciparum*. This protein is abundantly expressed in the red cell, released during rupture of infected red cells and can remain in the blood for up to 28 days after the initiation of anti-malarial therapy, making it an excellent biomarker for diagnosis [7-9].

A number of technical issues which limit the utility of malaria RDTs have been documented. An important one is their limited shelf life, due to the temperature sensitive capture and detecting antibody reagents [10,11]. As RDTs are increasingly being deployed in remote tropical areas, this problem could potentially lead to inaccurate diagnosis and misuse of drugs [12]. Therefore, newly and more stable antigen binders represent important objectives in improving malaria RDT performance.

The aim of this study was to isolate new monoclonal antibodies (mAbs) to PfHRP2 that may have better specificity and stability than those currently used in RDTs. To this end, a human single chain variable fragment (scFv) naïve library was screened to isolate unique binders specific for PfHRP2 using phage display technology. Antibody phage display has been extensively used for isolation of high affinity specific binders against unique antigens from different targets [13-17]. This technology was first introduced by Smith in 1985 and has been widely used in diagnostic and therapeutic applications as previously reviewed [18,19]. Such recombinant antibodies have been shown to be effective in targeting different biomarkers in various diseases. Over the past ten years, about 30% of human therapeutic antibodies that have reached clinical trials have been identified by phage display technology [20,21].

## Methods

### Malaria PfHRP2 antigen

A recombinant PfHRP2 malaria protein encoded by the DNA sequence of the *P. falciparum* line FCQ79 [22], fused with thioredoxin tag and expressed in *Escherichia coli* by DNA recombinant technology (Nelson Lee, unpublished) was used. This recombinant protein, rPfHRP2, has been shown to react with a set of PfHRP2-specific mouse monoclonal antibodies [23].

### Isolation of PfHRP2-specific scFv antibodies by phage display

The ‘Sheets’ naive human scFv phage display library, with a reported diversity of 6.7 × 10^9^ members isolated from peripheral blood lymphocyte cDNA from five different donors, was kindly provided by Prof James Marks (University of California, San Francisco, USA) [24].

Iterative rounds of biopanning were performed to isolate human scFv specific for rPfHRP2 from the amplified Sheets library. Briefly, two lots of 100 μg/mL purified thioredoxin (Sigma-Aldrich, USA) in phosphate buffered saline (PBS), and one lot of 100 μg/mL rPfHRP2 protein in PBS were incubated overnight at room temperature in immunotubes (Nunc Maxisorp; Denmark). The next day, the immunotubes were rinsed and blocked with 2% skimmed milk powder (Diploma, Australia) in PBS (MPBS) for 1 hour at room temperature. Phage particles (1.2 × 10^13^) were blocked in 2% MPBS for 1 hour at room temperature and then incubated for two sequential 1-hour incubations in the thioredoxin-coated immunotubes. The unbound phage were then transferred to the rPfHRP2-coated tube and incubated for a further 1 hour at room temperature. Unbound phage were removed by washing five times with 0.1% Tween 20 in PBS (PBST). Bound phage were eluted with 1 mL of 200 mM glycine at pH 2.5, and the eluate was immediately neutralized by adding 0.5 mL of 1 M Tris–HCl, pH 7.4. Eluted phage were infected into log phase XL1-Blue *E. coli* bacteria, and then amplified by growth in 50 mL of 2YT medium supplemented with 100 μg/mL ampicillin and 2% glucose. Phage were rescued by infection with 1 × 10^11^ pfu of M13K07 helper phage (NEB, USA), followed by overnight incubation at 30°C in 2YT medium supplemented with 100 μg/mL ampicillin and 30 μg/mL kanamycin. Rescued phage were concentrated from the culture supernatant by precipitation with 4% PEG 6000 and 0.5 M NaCl, then used for the next round of panning. In total, five rounds of selections were performed with antigen concentrations of 100 μg/mL for rounds 1 and 2, 50 μg/mL for round 3, 25 μg/mL for round 4 and, 12.5 μg/mL for round 5.

### Analysis of isolated clones

Following panning, the phage pool and isolated clones were evaluated for binding against recombinant PfHRP2 by polyclonal and monoclonal ELISA, respectively. Briefly, the binding reactivity of phage supernatant was tested in 96-well ELISA plates (Nunc Maxisorp, Denmark) coated with 10 μg/mL of rPfHRP2 and blocked with 2% MPBS. Signals were detected with horseradish peroxidase (HRP)-conjugated anti-M13 monoclonal antibody (GE Healthcare, Australia). The DNA sequence of positive clones was determined by Sanger sequencing in Applied Biosystems Sequencer Model 3100 using pHEN1 sequencing primers. After sequencing, the amino acid sequences were deduced and aligned using ClustalW; the three complementarity determining regions (CDRs) and four framework (FW) regions in each of variable heavy (VH) and variable light (VL) chain regions were identified using the IMGT V-Quest software and VBASE2 immunoglobulin database [25].

### Antibody reformatting

To reformat the scFv fragments to whole, fully human IgG, the variable regions for both the heavy and kappa light chains of the rPfHRP2 specific scFv clones D2 and F9 were PCR amplified from the phagemid vectors and cloned into SacI-linearized ReformAb vectors (Acyte Biotech, Australia) as previously described [26].

### Mammalian cell expression and purification

The expression and purification of mAbs D2 and F9 was undertaken as previously described [26]. Briefly, the heavy and light chain plasmids were co-transfected into suspension-adapted Chinese hamster ovary (CHO) cells after mixing of plasmids with polyethylenimine (PEI). After four hours, cells were diluted by doubling the total volume with CD-CHO media, and insulin-like growth factor 1 (IGF-1) (Novozymes, USA) was added at a concentration of 0.1 mg/L. The cultures were then transferred to a humidified incubator and maintained at 32°C and 7.5% CO_2_ for seven to 14 days with shaking (250 rpm). Antibodies were purified using Protein A affinity chromatography. Bound antibodies were eluted at low pH and then immediately buffer exchanged into PBS. Protein concentration was determined by absorbance at 280 nm, and used to determine the yield. The quality of purified monoclonal antibodies (D2 and F9) was determined by SDS-PAGE gel and size-exclusion high-performance liquid chromatography (HPLC) analysis.

### Antibody binding sensitivity determined by endpoint dilution ELISA

To understand the binding efficacy of these mAbs, a malaria PfHRP2-specific mAb C1-13 (kindly provided by Dr Martin Bubb, National Products Institute, South Africa) was used as positive control in this study. mAb C1-13 is a commercial mouse IgG1 mAb with binding specificity against PfHRP2, that has been reported in previous studies [23,27].

Microtitre plates (Greiner Bio-One, Germany) were coated with 100 μL of diluted rPfHRP2 protein (1 μg/mL) and blocked with 2% MPBS. After three wash steps with 0.1% PBST, the blocked wells were incubated with 100 μL of two-fold serial diluted mAbs D2 and F9, and control antibody C1-13, starting at 20 ng/mL for 1 hour at room temperature. Plates were washed with 0.1% PBST. One-hundred μL of secondary detection antibody was added, using goat anti-human antibody-conjugated HRP (Cat #:A8667) (Sigma-Aldrich; MO, USA), diluted 1:5,000 in 2% MPBS for D2 and F9 wells, or secondary goat anti-mouse antibody-conjugated HRP (Cat #: 115-035-003) (Jackson, USA), diluted 1:5,000 in 2% MPBS for C1-13 wells. Plates were incubated at room temperature for 1 hour. After washing with 0.1% PBST, 100 μL of freshly prepared OPD solution (Sigma, USA) was added to each well, and absorbance read at a wavelength of 405 nm using ELISA plate reader (Biotek Synergy HT, USA).

### Antibody binding specificity to rPfHRP2 and native proteins by Western blot

Saponin lysis was used to lyse the erythrocyte cell membranes, a process that leaves the cell membrane intact. *P. falciparum* culture was undertaken with the reference strain 3D7 in human O + blood (Red Cross Blood Service, Brisbane). Cultures were synchronized and harvested at 5–10% parasitaemia. The parasite sample was then washed and purified as described previously [28], and mixed 1:1 with 2× reducing SDS-PAGE sample buffer.

Following SDS-PAGE electrophoresis, the separated rPfHRP2 proteins (1 μg/mL), and native protein (1 μg/mL) were transferred to nitrocellulose membrane (Hybond-c Amersham Bioscience, UK). Uninfected red cells from the same lot were used as controls. Membranes were blocked in 5% MPBS and incubated for 1 hour at room temperature. After three wash steps with 0.1% PBST, 30 mL of test mAbs at a concentration of 10 μg/mL was incubated on the membrane at room temperature for 1 hour with gentle shaking. Following three wash steps with 0.1% PBST, membranes were incubated with secondary goat anti-human antibody-conjugated HRP (Cat#: A8667) (Sigma-Aldrich, USA) that was diluted 1:10,000 in 5% MPBS for mAbs D2 and F9 or goat anti-mouse antibody-conjugated HRP (Cat#: 115-035-003) (Jackson, USA) that was diluted 1:10,000 in 5% MPBS for mAb C1-13. The detection of HRP-conjugated secondary antibody was performed using ECL chemiluminescence detection kit (Amersham Bioscience, USA).

### Antigen sensitivity determined by ELISA

Microtitre plates (Nunc Maxisorp, USA) were coated with ten-fold serially diluted rPfHRP2 protein (100 μL/well), with a starting concentration of 1 μg/mL. Plates were then blocked in 2% MPBS and washed three times with 0.1% PBST. The primary test antibodies (D2, F9 and C1-13) were prepared at concentrations of 1 μg/mL and 5 μg/mL and 100 μL were added to corresponding wells and the detection of secondary antibody was undertaken as described above.

### Sensitivity determined by dot blot

10–15 μL of 1,000-fold serially diluted rPfHRP2 protein (starting at 1 μg/mL) was transferred to the nitrocellulose membrane (Hybond-C, Amersham Bioscience, UK) by vacuum aspiration. After blocking in 5% MPBS for 1 hour, the membranes were cut into small strips and placed into corresponding tubes which contained 30 mL of appropriate test antibodies (D2, F9 and C1-13) at concentrations of 1 μg/mL and 5 μg/mL. The blot images were developed as described above.

### Sandwich configuration studies

Microtitre plates coated with 100 μL of capture antibodies D2, F9 or C1-13 (10 μg/mL) were blocked by 300 μL of 5% MPBS. Ten-fold serially diluted rPfHRP2 protein dilutions (starting at 1 μg/mL) were transferred to the corresponding wells and incubated for 1 hour. After three washes with PBST, bound antigen was detected by incubation with 100 μL of mAbs D2 or F9 (10 μg/mL) (for plates coated with C1-13), or C1-13 (10 μg/mL) (for plates coated with D2 and F9). After incubation at room temperature for 1 hour, plates were washed as described above. Detection was achieved using 100 μL of mouse anti-human antibody-conjugated HRP (Sigma-Aldrich, MO, USA) for wells with mAbs D2 and F9 detection antibodies or goat anti-mouse antibody conjugated HRP for wells using C1-13 detecting antibody in 2% MPBS as described above.

### Heat lability determined by differential scanning calorimetry

The melting point of purified mAbs D2, F9 and C1-13 was measured by differential scanning calorimetry (DSC) using a NanoDSC Microcalorimeter (TA Instrument, USA) at an antibody concentration of 1.0 mg/mL. The antibodies were scanned at a rate of 1°C per minute from 25°C to 110°C, and analysed using DSC software.

### Binding efficacy of mAbs stored at different temperatures by dot blot analysis

Ten to 15 μL of two-fold serially diluted rPfHRP2 protein (starting at 1 μg/mL) was transferred to nitrocellulose membrane by vacuum aspiration. After blocking in 5% MPBS at room temperature for 1 hour, membranes were cut into small strips and placed into corresponding tubes which contained 30 mL of test D2, F9 and control C1-13 antibodies (10 μg/mL) that had been separately incubated at 25°C for seven days, and 37°C for 30 days. The secondary antibody incubation and detection were undertaken as described above.

### Epitope mapping

A total of 171 different peptides with length of 15 amino acid residues designed to represent different sequences and regions of PfHRP2 were used in epitope mapping as previously described [23].

### Antibody binding affinity determined by Octet

The binding affinity of human mAbs with rPfHRP2 was determined using anti-human IgG capture (AHC) biosensors in the Octet Red Instrument (Fortebio, USA). Purified mAbs (20 μg/mL) and ten-fold serially diluted rPfHRP2 (starting at 50 μg/mL) were prepared in assay buffer (10 mM phosphate, 150 mM NaCl, 0.02% Tween 20, 0.05% sodium azide, and 1 mg/mL bovine serum albumin (BSA), pH7.4). Briefly, a 96-well black microtitre plate (Perkin Elmer, USA) was prepared and 200 μL of each sample and buffer were loaded into corresponding wells. After inserting the hydration plate, both hydrated biosensor assembly and sample plate were equilibrated prior to the assay starting. The default settings with minor modifications were used for the assays. The data acquisition at 1 minute/six biosensors, flow rate was at 200 mm/second, precision was at <10% coefficient of variations (CVs), and standard curve fit was calibrated at four-parameter logistic (4-PL) model. The binding rate analysis was performed by Octet Data Analysis software version 6.1.

## Results

### Biopanning and selection of anti-PfHRP2 scFv clones

**Figure 1:**
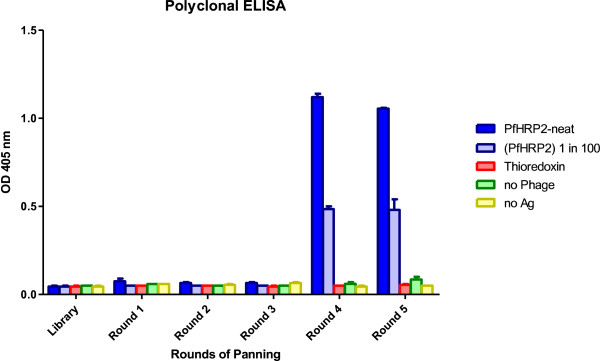
**Polyclonal phage ELISA.** The diagram shows an increase in ELISA absorbance values in rounds 4 and 5 phage pools against neat, and 1 in 100 diluted rPfHRP2 proteins. Immobilized thioredoxin protein, no phage, and uncoated microtitre plate wells were used as negative controls.

A naïve human scFv antibody phage display library was screened against the immobilized malaria antigen rPfHRP2. After five rounds of biopanning, the phage pool showed significant enrichment of binders to rPfHRP2 by polyclonal phage ELISA analysis (Figure 1). Eighty-four clones were randomly picked from the fifth round phage pool and 81 showed strong binding in a monoclonal phage ELISA assay.

Twenty-four positive clones and one negative clone were chosen for further analysis. PCR-amplified inserts with an expected size of ~500 bp were achieved in all clones except one. Of these clones, 13 were found to have an identical sequence. An additional four clones also appeared to have this same sequence, although the sequence data was incomplete. Thus, the population contained one highly dominant clone. An additional sequence was represented by two clones: F9 and B6. One other clone contained an out-of-frame coding region and corresponded with the ELISA negative clone. Two clones (D2 and F9) representing the two unique sequences were selected for further analysis. Sequence analysis of the VH and VL fragments revealed that both clones belonged to human immunoglobulin IgHV3 and IgKV1 families, respectively.

### Antibody expression

**Figure 2:**
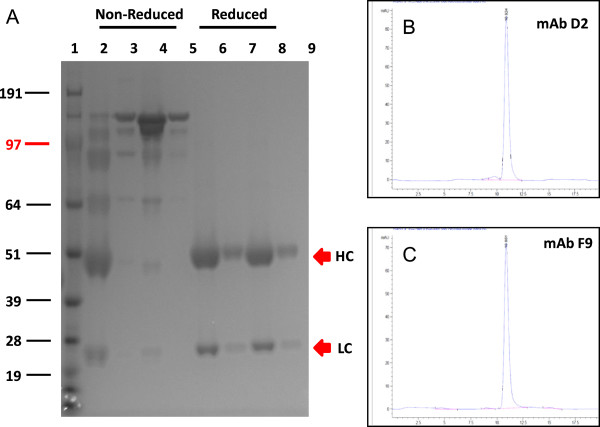
**Analysis of purified recombinant mAbs D2 and F9 on 4-12% SDS-PAGE.** The gel was stained with Coomassie Blue R250 **(A)**. Lane 1: SeeBlue Plus2 Pre-Stained Standard (Invitrogen); Lanes 2 and 3: non-reduced mAb D2; Lanes 4 and 5: non-reduced mAb F9; Lanes 6 and 7: reduced mAb D2; Lanes 8 and 9: reduced mAb F9, at neat, and 1 in 10 dilutions. Analytical size exclusion chromatography of protein-A purified recombinant mAbs D2 and F9 at 280 nm are shown in panels **B** and **C**, respectively.

The D2 and F9 scFv fragments were reformatted to whole mAbs, expressed in CHO cells and purified by Protein A chromatography [26]. SDS-PAGE analysis of the purified mAbs showed the whole intact mAb in non-reducing conditions (~150 kDa), and a 50 kDa heavy chain and 25 kDa light chain in reducing conditions. Size exclusion HPLC showed no significant aggregation or degradation (Figure 2).

### Characterization of antibody

**Figure 3:**
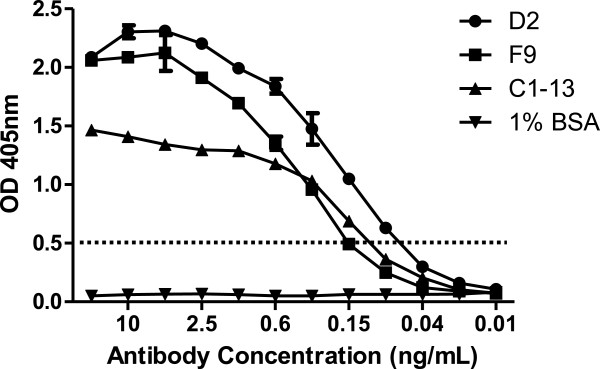
**Titration of mAbs D2, F9, and C1-13 against 0.1 μg rPfHRP2 protein.** The antibodies were prepared in two-fold serial dilution, from 20 ng/mL in ELISA assay. 1% BSA protein was used as a negative control. The dotted line represents cut-off OD absorbance at 0.5.

ELISA was employed to define the titre (endpoint dilution) of each mAb against 100 μg/ml of rPfHRP2 (Figure 3). When the cut-off absorbance is set at 0.5, mAb D2 had the highest end-titre sensitivity at 0.06 ng/mL, followed by mAb C1-13 at 0.1 ng/mL and mAb F9 at 0.15 ng/mL. No reactions were observed for these mAbs in ELISA assays and Western blot when rPfHRP2 was replaced by 1% BSA.

**Figure 4:**
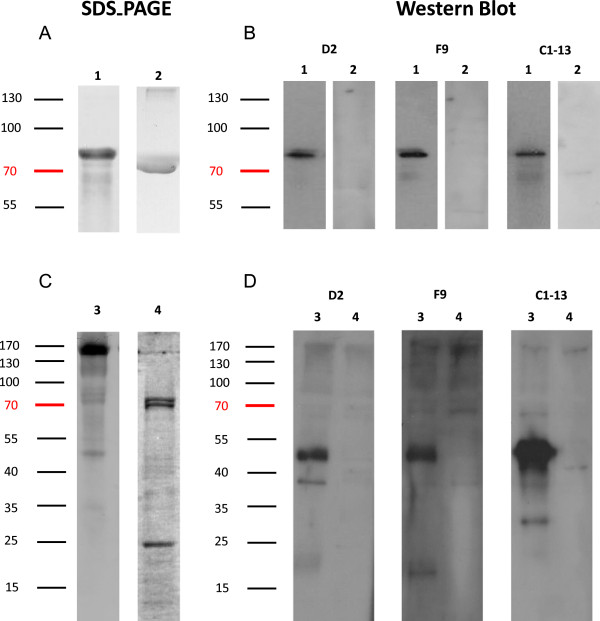
**Western blot analysis for the binding specificity of mAbs against recombinant and native PfHRP2 proteins. A**: Separation of recombinant PfHRP2 and 1% BSA proteins in 10% reduced SDS-PAGE, and stained with Coomassie Blue; **B**: Hybridization of corresponding recombinant mAbs D2, F9, and C1-13 to rPfHRP2 (1 μg/mL) and 1% BSA in Western blot. **C**: Separation of native malaria parasite protein and uninfected RBC by10% SDS-PAGE, stained with Coomassie Blue. **D**: hybridization of corresponding recombinant mAbs D2, F9, and C1-13 to malaria native protein (1 μg/mL) and uninfected RBC protein (1 μg/mL)in Western blot. Lane 1: recombinant HRP2 protein; Lane 2: negative control 1% BSA. Lane 3: *P. falciparum*-infected RBC protein; Lane 4: negative control uninfected RBC protein. Molecular weights are shown at the left corner of the diagram.

**Figure 5:**
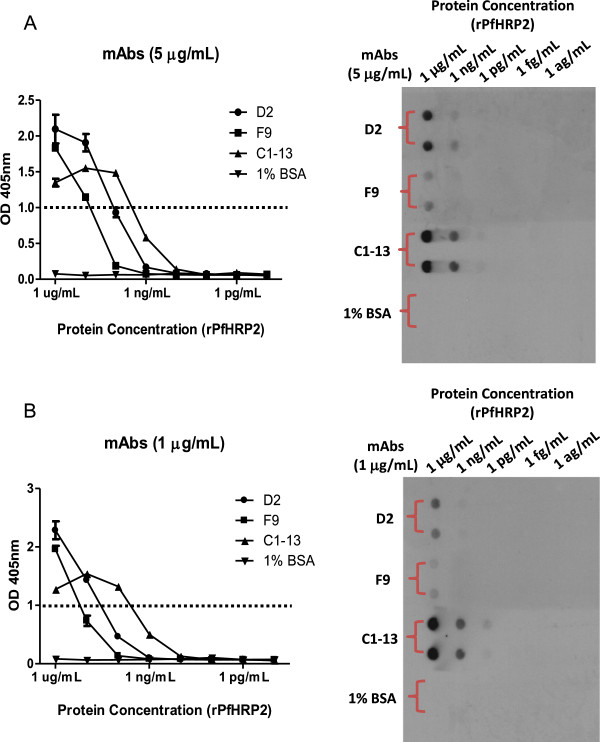
**Sensitivity of mAbs D2, F9 and C1-13 against rPfHRP2.** The binding efficacy of mAbs against serially diluted recombinant PfHRP2 is determined by ELISA (Left panel) and dot blot (Right panel). **A**: 5 μg/mL of mAbs; **B**: 1 μg/mL of mAbs. Dot line represents cut-off OD absorbance at 1.0.

**Figure 6:**
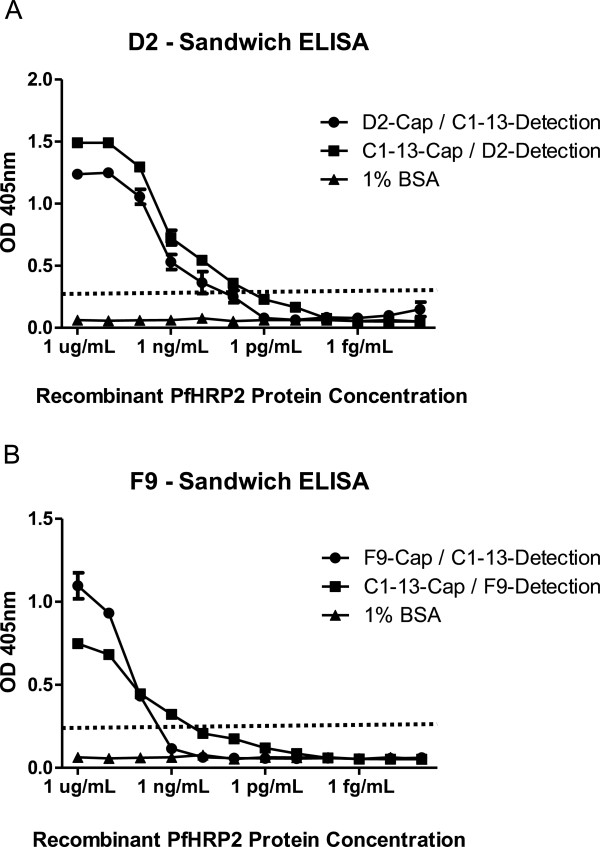
**Binding reactivity of mAbs D2 and F9 and mAb C1-13 to rPfHRP2 protein.** mAbs D2 **(A)** and F9 **(B)** were used as capture antibody (Cap) or detection antibody (Detection) in combination with mAb C1-13 in sandwich ELISA. The dotted line represents cut-off OD absorbance at 0.25.

The specificity of mAbs against recombinant and native malaria proteins was evaluated by Western blot analysis (Figure 4). Both mAbs D2 and F9 reacted to rPfHRP2, showing a single band at 82 kDa, the correct size for the thioredoxin-fused protein. For detection of native parasite proteins from blood stage infection, *P. falciparum*-infected red blood cells were tested in Western blot. The results show that all mAbs were capable of recognizing native PfHRP2 protein at ~50 kDa, with such band was not detected from uninfected RBC protein lane. No cross-reactivity with the other two malaria biomarkers, namely *P. falciparum* lactate dehydrogenase (PfLDH) and *Plasmodium vivax* aldolase (PvAldolase) was observed on Western blot (Additional file 1) or ELISA (Additional file 2).The sensitivity of detecting rPfHRP2 protein by mAbs was investigated by ELISA and dot blot analysis (Figure 5). The ELISA data indicated a difference in sensitivity between three mAbs when comparing the detection limit against serial diluted rPfHRP2. While 5 μg/mL of mAbs applied, mAb C1-13 had the highest sensitivity, with an OD value of 1, the detection achieved 5 ng/mL of rPfHRP2, followed by mAbs D2 and F9 at 10 ng/mL and 100 ng/mL of rPfHRP2, respectively (Figure 5A). However, sensitivity fell when less concentrated antibodies were applied (1 μg/mL) (Figure 5B). These results were consistent with results of the semi-quantitative studies carried out by dot blot. Compared to the commercial antibody C1-13, the sensitivity of both antibodies D2 and F9 was lower. Detection limits were ten times (D2) and 100 times (F9) lower than for C1-13.To determine the accuracy and linearity of rPfHRP2 detection, mAbs of D2 and F9 were each designed as capture or detection antibodies in sandwich ELISA, in combination with mAb C1-13. The mean reactivity for duplicate measures of serially diluted rPfHRP2 protein from 1 μg/mL to 0.01 fg/mL detected by mAbs D2 and F9 are represented in Figure 6. From the results, both antibodies D2 and F9 showed binding to rPfHRP2 protein in heterologous configurations with mAb C1-13. However, the sensitivity of heterologous antibodies varied despite the use of the same mAbs D2 or F9 as either capture or detection antibodies. When using an OD cut-off value of 0.25, the detection limit for D2-Cap/C1-13-Detection was at 50 pg/mL, whereas C1-13-Cap/D2-Detection was at 1 pg/mL (Figure 6A). The configuration of mAbs F9 and C1-13 influenced different sensitivity. The lowest detection limit for F9-Cap/C1-13-Detection was 5 ng/mL, and C1-13-Cap/F9-Detection was ~0.5 ng/mL at the OD cut-off value of 0.25 (Figure 6B). Overall, the configuration of C1-13-Cap/D2-Detection was the most sensitive for detection of rPfHRP2 protein by ELISA. Neither heterologous mAbs D2 nor F9 reacted to 1% BSA protein.

### Heat lability of antibodies

The melting point value of mAbs was determined using DSC analysis (Additional file 3). Two melting point peaks (Tm) were detected for the mAbs, namely 72°C and 82°C for mAb D2, and 73°C and 80°C for mAb F9. Only one melting point of 72°C was observed for the control antibody C1-13. The precipitation of mAbs D2 and F9 at high temperatures was indicated by a sharp peak decline and negative Cp values. This phenomenon was not observed with mAb C1-13, where the Cp values returning to baseline following Tm peak.

After incubation at different temperatures for different durations, the heat stability of two recombinant mAbs were determined by reacting to two-fold serially diluted rPfHRP2 proteins immobilized on nitrocellulose membrane using dot blot (Additional file 4). mAb C1-13 had previously been shown to retain its reactivity after incubating at 37°C for 90 days [23]. After incubation at 25°C for seven days, the binding ability of mAbs D2, F9 and C1-13 was still maintained (Additional file 4A). The heat stability of mAbs was different; mAb D2 retained its binding sensitivity at 37°C compared to 25°C, whereas loss of sensitivity at 37°C was observed in mAbs C1-13 and F9 (Additional file 4B).

### Epitope mapping

In initial epitope mapping, both mAbs D2 and F9 gave positive reactions to a range of different peptides. mAb D2 recognized the predominant motif AHHAADAHHA as its major epitope with possible substitutions of serine in the fifth amino acid, a similar result to the major epitope recognized by mAb C1-13 [23]. In contrast, the major epitope recognized by mAb F9 was one amino acid shorter, lacking an alanine at the end, resulting in AHHAADAHH.

### Evaluation of binding affinity of mAbs

A kinetic study was performed to determine the binding affinity of mAbs to serially diluted rPfHRP2 proteins from 610–0.0061 nM (Additional file 5). Of the three mAbs, the highest association constant (k_a_) value was displayed by F9 at 1.80 × 10^6^ Ms^−1^ (Additional file 5B), whereas D2 was at 8.50 × 10^5^ Ms^−1^ (Additional file 5A). In comparison, C1-13, previously measured by Biacore (Lee *et al.* unpublished) has an association value of 3.60 × 10^5^ Ms^−1^ (Lee *et al.* unpublished). The dissociation constant (k_d_) value of F9 was however the lowest value at 7.66 × 10^−5^ s^−1^ compared to D2 (2.59 × 10^−4^ s^−1^) and C1-13 (3.72 × 10^−5^ s^−1^).

To understand the binding affinity value, the K_D_ was calculated by k_d_/k_a_. This showed that mAb F9 had the strongest binding affinity to rPfHRP2 protein, with a K_D_ value of 4.27 × 10^−11^ M (Table 1), followed by control mAb C1-13 at 1.03 × 10^−10^ M and mAb D2 at 3.05 × 10^−10^ M.

**Table 1:** Kinetics determination of mAbs D2 and F9

**mAb**	**k**_**a **_**(1/Ms)**	**k**_**d **_**(1/s)**	**K**_**D **_**(M)**
D2	8.50 × 10^5^	2.59 × 10^−4^	3.05 × 10^−10^
F9	1.80 × 10^6^	7.66 × 10^−5^	4.27 × 10^−11^
C1-13	3.60 × 10^5^	3.72 × 10^−5^	1.03 × 10^−10^

Association (k_a_), dissociation rates (k_d_), and equilibrium constants (K_D_) of mAbs D2, F9, and C1-13 against rPfHRP2. The analysis for mAbs D2 and F9 were undertaken using the Octet Red platform, whereas mAb C1-13 was determined by BIAcore.

## Discussion

Since the introduction of lateral flow immunochromatographic assays for detection of malaria about 20 years ago [29,30], RDTs have played an increasing role in improving the quality of case management for malaria and assist the reduction of unnecessary anti-malarial drug usage [31,32]. The specificity, sensitivity, affinity, and heat stability of antibodies are important determinants of the quality and reliability of a RDT.

Current malaria RDTs are developed in formats such as dipstick, strip, card, pad, well, or cassette [33]. Three types of antibodies, including signal, capture and control antibodies are generally incorporated in the device to render indication of a complex antibody-antigen interaction [10,33]. However, variation of sensitivity has been reported in many current commercial available RDTs [34]. The degradation of antibody performance with long exposure to high temperature has led to a decrease in operational performance [35]. To address this problem, a selection of quality mAbs with high affinity, specificity and heat stability to target antigen is important to improve malaria diagnostic in tropical regions.

The aim of this study was to generate new mAbs and assess their binding capability against malaria PfHRP2 antigen. The Sheets naive human scFv phage display library has been shown to contain binders to a wide range of different antigens, with affinities achieved in subnanomolar range [24]. This provided a good rationale for using this library to isolate novel scFv fragments targeting parasite biomarkers. Following five rounds of biopanning of the Sheets library, two unique scFv clones with specificity to rPfHRP2 were isolated. These clones were reformatted to whole IgG1 to allow comparison with a commercially available mouse antibody against PfHRP2.

Most antibodies deployed currently in commercial malaria RDT devices were produced in the early 1990s. Due to commercial consideration, details such as mAbs isotype, subclasses, epitopes targeted, and laboratory of origin are either rarely published or incompletely documented by manufacturers [22]. Herein, the performance of the two newly isolated human mAbs produced in this study was compared with commercially available mouse mAb C1-13, which was shown to have the highest sensitivity and specificity among a panel of monoclonal IgGs targeting PfHRP2 [23].

Both mAbs D2 and F9 exhibited strong binding specificity and sensitivity against both recombinant and native PfHRP2, as shown by ELISA and Western blot (Figures 3 and 4). Like C1-13, both reformatted mAbs recognized rPfHRP2 as a single band on Western blot, suggesting that the specificity of mAbs D2 and F9 was not affected by reformatting. In extracts from infected red blood cells, all mAbs recognized a major band of 50 kDa, the size of native PfHRP2. The presence of multiple bands on Western blot is not uncommon when anti-PfHRP2 mAbs are used against native parasite proteins, because *P. falciparum* has several histidine-rich proteins [36]. One of these is PfHRP3, a protein highly similar to PfHRP2 in amino acid sequence with a molecular weight of approximately 35–37 kDa [37]. Antibodies against PfHRP2, such as mAb C1-13, have been reported to bind to PfHRP3 [23]. However, only two bands (50 kDa and 37 kDa) were recognized by mAb D2, while one band (50 kDa) by mAb F9, suggesting these mAbs may have higher specificity than C1-13 [38,39].

When D2 and F9 antibodies were tested either as capture antibody or detection antibody by partnering with mAb C1-13 (Figure 6), both antibodies showed no significant loss of sensitivity of binding in the sandwich format. The detection sensitivity was best when mAb C1-13 was used as capture antibody although different secondary antibodies used for detection may contribute to the detection sensitivity. As an extensive number of repeated epitopes exist in PfHRP2 [6], epitope numbers are not likely contributing to the difference in binding of mAbs [40]. The difference may result from better binding affinity of mAb C1-13 to the double HHA (amino acid residues) sequence on antigenic site of PfHRP2 epitopes. However, mAb C1-13 was shown to possess lower affinity than mAb F9. Therefore the causes contributing the binding behaviour of these mAbs remain unclear.

The Octet Red® system was chosen to examine, in real time, the rate of association (binding) and dissociation (unbinding) of mAbs D2 and F9 to rPfHRP2 protein. The mAb F9 showed the highest binding affinity (K_D_ of 40 pM), although it had a weaker sensitivity in the ELISA screening (Figure 5). This was followed by mAbs C1-13 (0.1 nM) and D2 (0.3 nM). These are very strong affinities, despite being isolated from a naive library, which may in part be explained by the nature of the epitope. The optimal epitope recognized by mAb D2 was identical to that recognized by mAb C1-13, namely AHHAADAHHA. Despite having the same epitope, mAbs C1-13 and D2 are able to simultaneously bind to PfHRP2 in a sandwich ELISA, since this epitope is present in many repeats along the PfHRP2 sequence as evidenced by the large number of analysed peptides from PfHRP2 that were able to bind these mAbs (41-60%) [23]. The repetitive nature of this epitope contributes to the very slow dissociation rates of these mAbs, as the antigen is providing high avidity, and consequently an apparent strong affinity. These factors are contributing to the poor fit of a kinetic binding model, which assumes a 1:1 interaction, shown in Additional file 5, so the kinetic values are an estimate only.

This strong apparent affinity is ideal for RDTs as mAbs that have a faster rate of association and slower rate of disassociation (a low k_d_ value) would be considered to give a greater binding ability to target biomarker. Thus, the kinetic properties of mAbs D2 and F9 make them potential candidates for malaria RDTs, and are at least comparable to the commercially available mAb C1-13, which has been shown to be superior to PfHRP2-specific IgM isotype antibodies [23].

Another aspect that important for reagents used in RDTs in the tropical and subtropical world is their ability to withstand high ambient storage temperatures. The experiment identified 72°C as the melting point for mouse mAb C1-13, 82°C and 80°C for D2 and F9, respectively (Additional file 3). When temperature gradually increased (>82°C), protein precipitation was not observed in control mAb C1-13 but was for both mAbs D2 and F9. Peaks in the readout trace may correspond to different parts of the mAb (eg the Fab region and Fc region) unfolding. According to literature, the CH2 domain of an antibody tends to unfold earlier, followed by the Fab and the CH3 domains. The thermal transition of the Fab domain commonly occurs between the CH2 and CH3 domains or overlapping with either one of this domain in a monoclonal antibody [41]. Furthermore, major difference in the amino acid composition between mouse and human immunoglobulins is another likely factor that resulted in these mAbs having different thermal stability [42-44]. The non-precipitation of mAb C1-13 indicated by Cp values returning to baseline shows this mAb possesses reversibility, which might not be found in the human mAbs D2 and F9. Due to lack of information about this commercial mAb, the complete amino acid composition and the production method were not available. However, this varying melting temperature may be influenced by thermodynamic interactions within the antibody domains, and conformational changes in human and mouse mAbs that correspond to the aggregation and precipitation of the unfolded protein [45,46].

In terms of stability, the unfolding of the human and mouse IgG1 at pH 5.5 generally present two transitions, with melting temperature around 70°C and 82°C. The unfolding of Fab fragment normally occurs at lower temperatures 70°C, whereas 82°C for Fc fragment [41]. Based on this finding, it may be speculated that the first and second unfolding events in the human mAbs are analogous with mouse mAbs. Therefore, human mAbs D2 and F9 may be expected to share a similar stability with mouse mAb C1-13 although only one peak was observed in mouse antibody. In addition to different melting profiles, the sequence of the variable domains is another factor potentially affecting the stability of the Fab fragment from either human or mouse [47]. Overall, the observation of melting point for mouse mAb C1-13, and human mAbs D2 and F9 suggested the mAbs produced in this study are more heat stable (by DSC analysis), but in practical terms when comparing stability at 25°C and 37°C all three mAbs exhibit similar stability.

In summary, phage display is a promising tool that allows the selection of new binders by undergoing iterative rounds of biopanning. In this study, two scFv clones isolated from human scFv naïve library were shown to have high affinity and specificity against PfHRP2 proteins. The binding efficacy of these recombinant mAbs was demonstrated to be comparable to commercial mAb C1-13. However, this system has some disadvantages, including duration of expression and expense of the eukaryotic expression system that may inhibit the use of the current system for manufacture of mAbs for malaria RDTs. Therefore, alternative expression method may be preferable. In addition, false positive results that occurred between native human RBC with anti-human antibodies, suggesting human Fc region might not be an ideal detection region for mAb recognition. Thus, tagging of the mAb, for example by biotinylation, may potentially reduce the cross-reactivity problem.

## Conclusions

Five rounds of biopanning resulted in selection of two high affinity clones from a naïve library. Recombinant mAbs D2 and F9, expressed by mammalian cells, are high quality mAbs which exhibited excellent binding capability to rPfHRP2. These antibodies were also demonstrated to have specificity and sensitivity comparable to commercial mAb C1-13 but with potentially better thermostability. Therefore, the mAbs produced in this study may be suitable for evaluation by undergoing field test in different malaria endemic regions in a prototype test kit.

## Abbreviations

BSA: Bovine serum albumin; CDRs: Complementarity determining regions; CV: Coefficient of variation; FW: Framework; HPLC: *High-performance liquid chromatography*; HRP: Horseradish peroxidase; IgG: Immunoglobulin G; mAb: Monoclonal antibody; PfHRP2: *P. falciparum* histidine-rich protein 2; RBC: Red blood cell; RDTs: Rapid diagnostic tests; rPfHRP2: Recombinant histidine-rich protein 2; scFv: Single chain variable fragment; VH: Variable heavy; VL: Variable light.

## Competing interests

The authors declare that they have no competing interests.

## Authors’ contributions

All authors made contribution to this study. HL and MJ conceived the general project study. JM, QC and SM coordinated the project and led manuscript writing. All authors have read and approved the final manuscript.

## Supplementary Material

Additional file 1 **Cross-reactivity determination of recombinant mAbs against three different malaria biomarkers in Western blot analysis.** Cross-reactivity determination of recombinant mAbs against three different malaria biomarkers in Western blot analysis. Lane 1: rPfHRP2; Lane 2: rPfLDH; Lane 3: rPvAldolase. The molecular weight and purity of each malaria biomarker is shown in panel A. The reactivity of mAbs D2, F9, and C1-13 are represented by panel B, C, and D, respectively. Click here for file

Additional file 2 **Cross-reactivity determination of recombinant mAbs against three different malaria biomarkers in ELISA analysis.** The reactivity of mAbs D2, F9, and C1-13 are represented by panel A, B, and C, respectively. Click here for file

Additional file 3 **The melting point for three mAbs in PBS pH 7.2 buffer by DSC analysis.** Calculated Tms for 1 mg/mL mAb C1-13 (purple) was 72°C; 1 mg/mL mAb D2 (green) had 2 peaks at Tms of 72°C and 82°C; and 0.5 mg/mL of mAb F9 (magenta) had two peaks at Tms of 73°C and 80°C. mAbs D2 and F9 precipitated at high temperatures indicated by sharp peak decline and negative Cp values. Control mAb C1-13 did not precipitate indicated by Cp values returning to baseline following Tm peak. Click here for file

Additional file 4 **Determination of heat stability at different temperatures and durations. **Dot blot was used to determine the heat stability of mAbs on a nitrocellulose membrane immobilized with two-fold serially diluted rPfHRP2 (starting at 1 μg/mL). The tested mAbs were incubated at 25°C for 7 days (A) and, at 37°C for 30 days (B) prior to hybridize to rPfHRP2 proteins. Click here for file

Additional file 5 **Determination of mAbs affinity by Octet sensogram.** Sensogram showing two mAbs binding to serially diluted rPfHRP2 proteins from 600 nM to 0.0061 nM. mAb D2 is represented in panel A. mAb F9 is represented in panel B. Coloured lines represent the binding interactions of mAb to different concentration of rPfHRP2. Red lines represent the statistical fitting of curves. Click here for file
