# Supplementary figures and images for: Production and characterization of specific monoclonal antibodies binding the Plasmodium falciparum diagnostic biomarker, histidine-rich protein 2
Source: Malar J. 2014 Jul 18;13:277. doi: 10.1186/1475-2875-13-277 (PMC4120728; doi:10.1186/1475-2875-13-277)

Supplement 1: Western blot

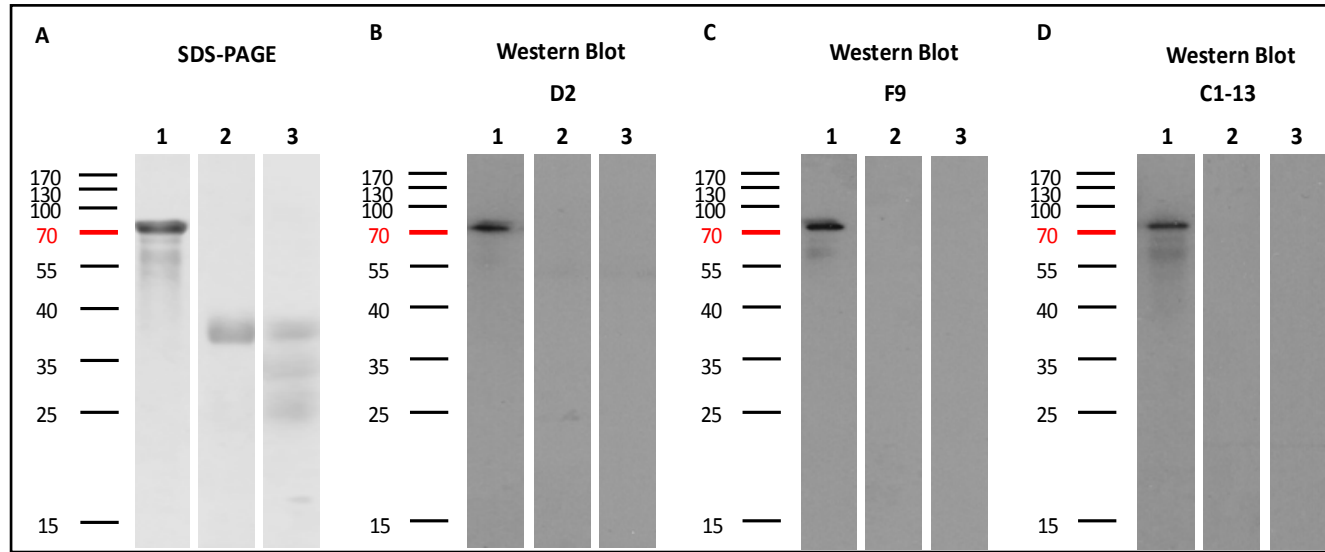

Supplement: Additional file 1 — Cross-reactivity determination of recombinant mAbs against three different malaria biomarkers in Western blot analysis. Cross-reactivity determination of recombinant mAbs against three different malaria biomarkers in Western blot analysis. Lane 1: rPfHRP2; Lane 2: rPfLDH; Lane 3: rPvAldolase. The molecular weight and purity of each malaria biomarker is shown in panel A. The reactivity of mAbs D2, F9, and C1-13 are represented by panel B, C, and D, respectively. [file 1475-2875-13-277-S1.pdf]

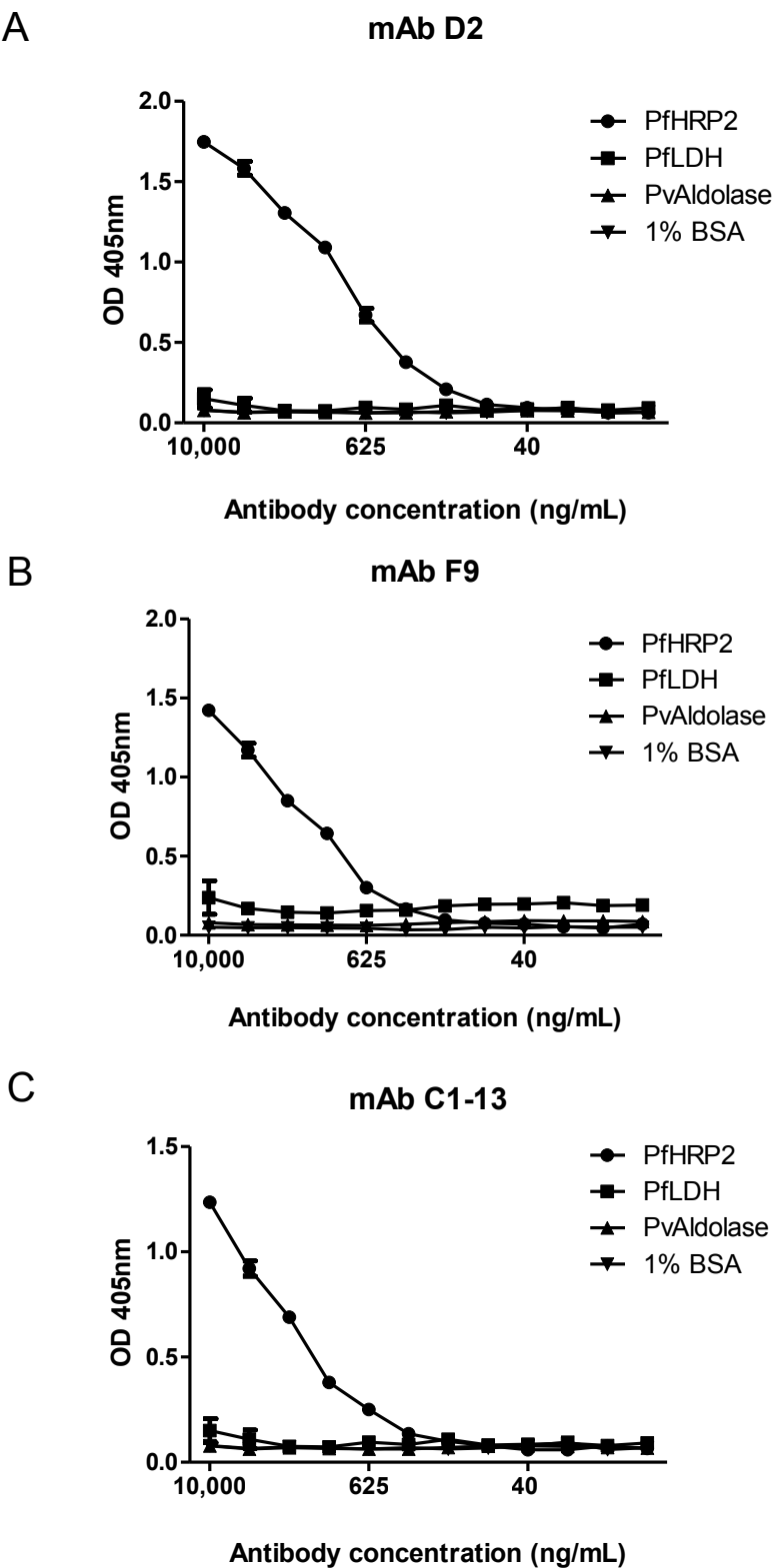

Supplement: Additional file 2 — Cross-reactivity determination of recombinant mAbs against three different malaria biomarkers in ELISA analysis. The reactivity of mAbs D2, F9, and C1-13 are represented by panel A, B, and C, respectively. [file 1475-2875-13-277-S2.pdf]

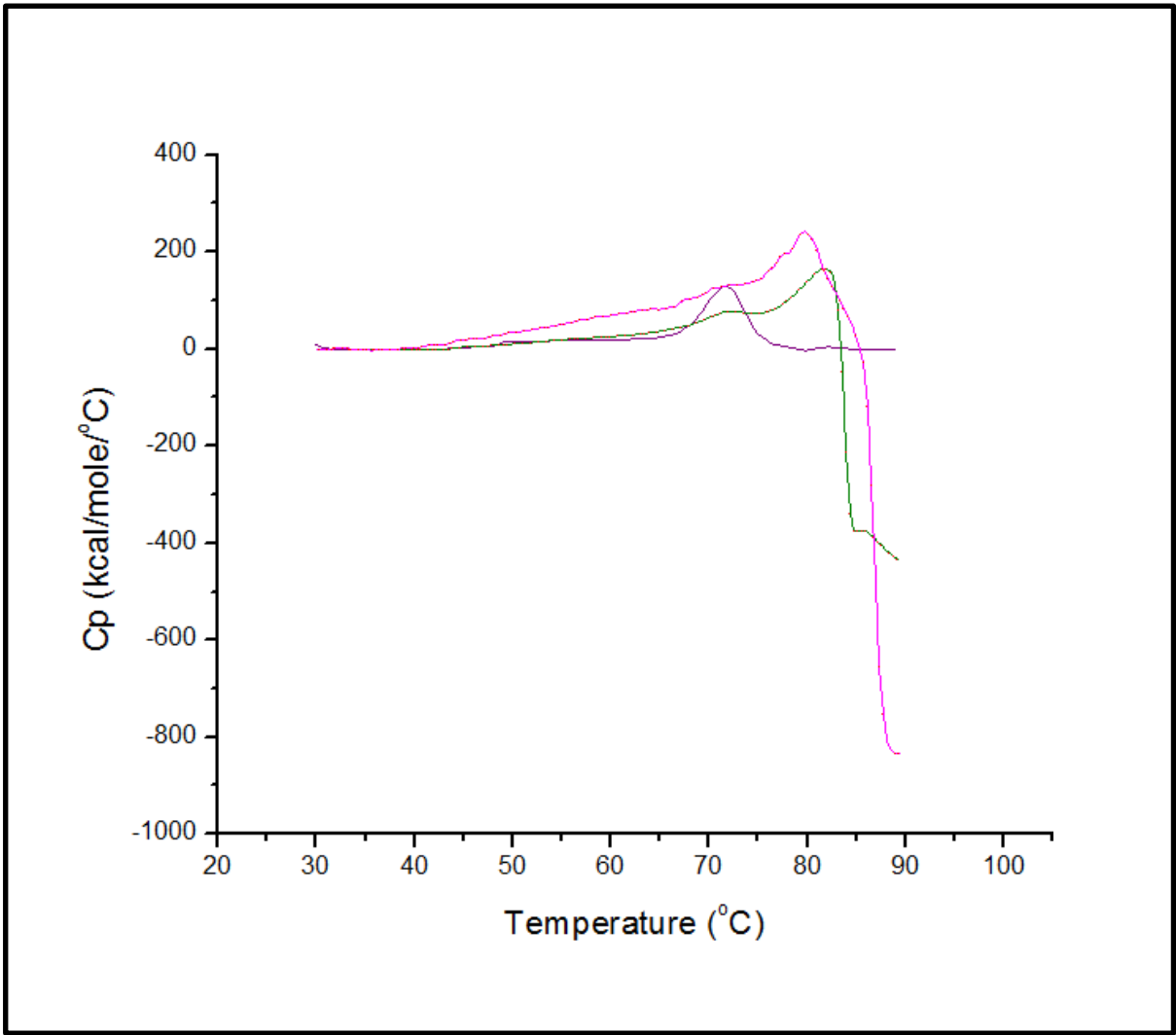

Supplement: Additional file 3 — The melting point for three mAbs in PBS pH 7.2 buffer by DSC analysis. Calculated Tms for 1 mg/mL mAb C1-13 (purple) was 72°C; 1 mg/mL mAb D2 (green) had 2 peaks at Tms of 72°C and 82°C; and 0.5 mg/mL of mAb F9 (magenta) had two peaks at Tms of 73°C and 80°C. mAbs D2 and F9 precipitated at high temperatures indicated by sharp peak decline and negative Cp values. Control mAb C1-13 did not precipitate indicated by Cp values returning to baseline following Tm peak. [file 1475-2875-13-277-S3.pdf]

**A** **mAbs incubated at 25 °C for 7 days**

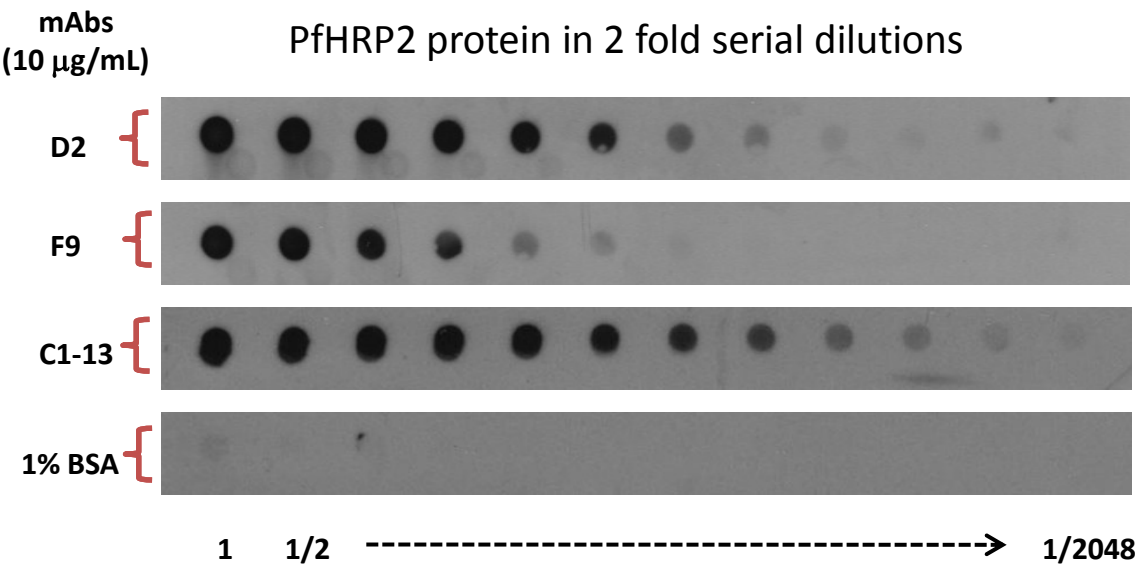

**B** **mAbs incubated at 37 °C for 30 days**

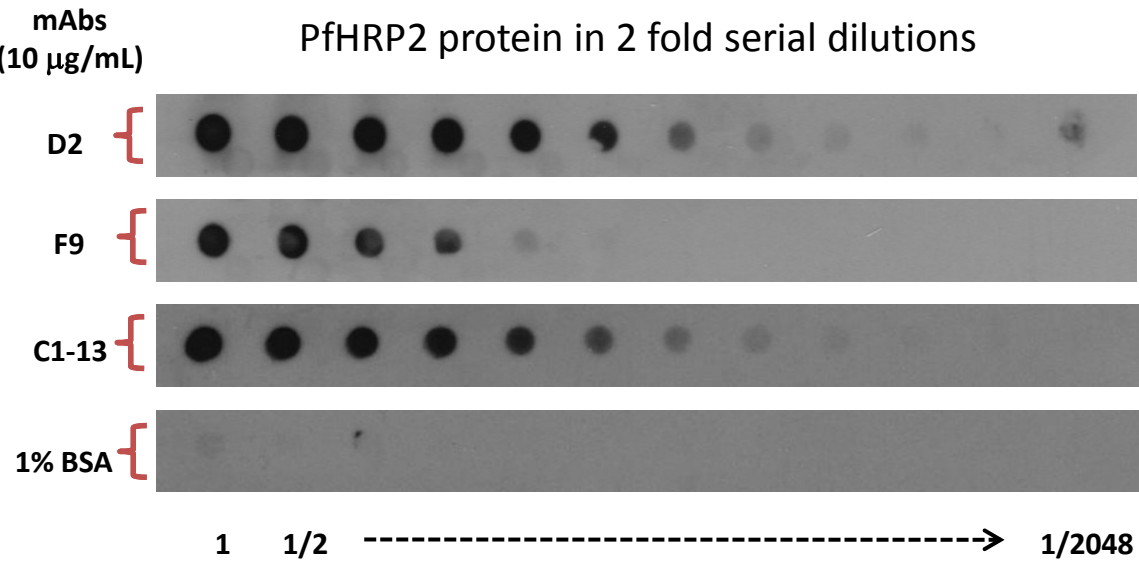

Supplement: Additional file 4 — Determination of heat stability at different temperatures and durations. Dot blot was used to determine the heat stability of mAbs on a nitrocellulose membrane immobilized with two-fold serially diluted rPfHRP2 (starting at 1 μg/mL). The tested mAbs were incubated at 25°C for 7 days (A) and, at 37°C for 30 days (B) prior to hybridize to rPfHRP2 proteins. [file 1475-2875-13-277-S4.pdf]

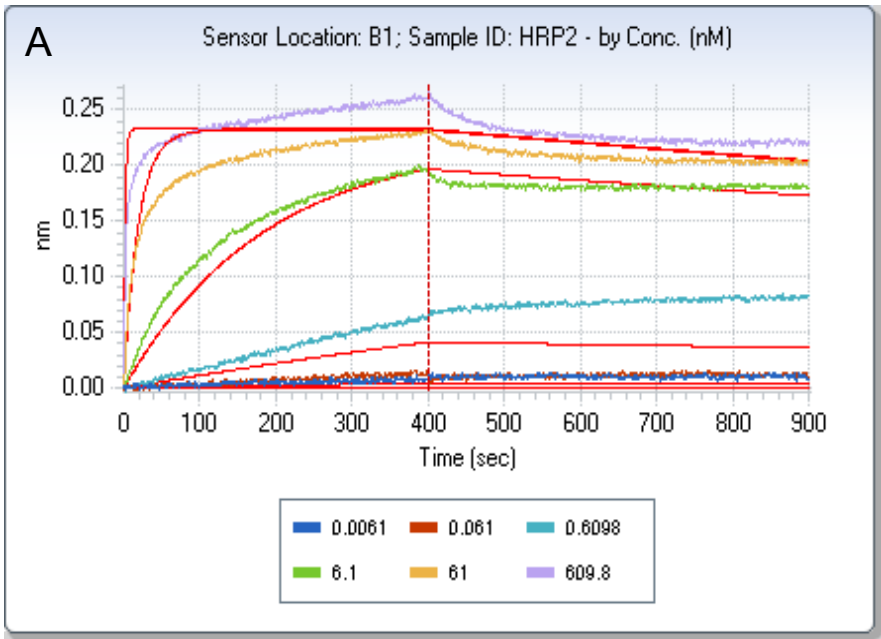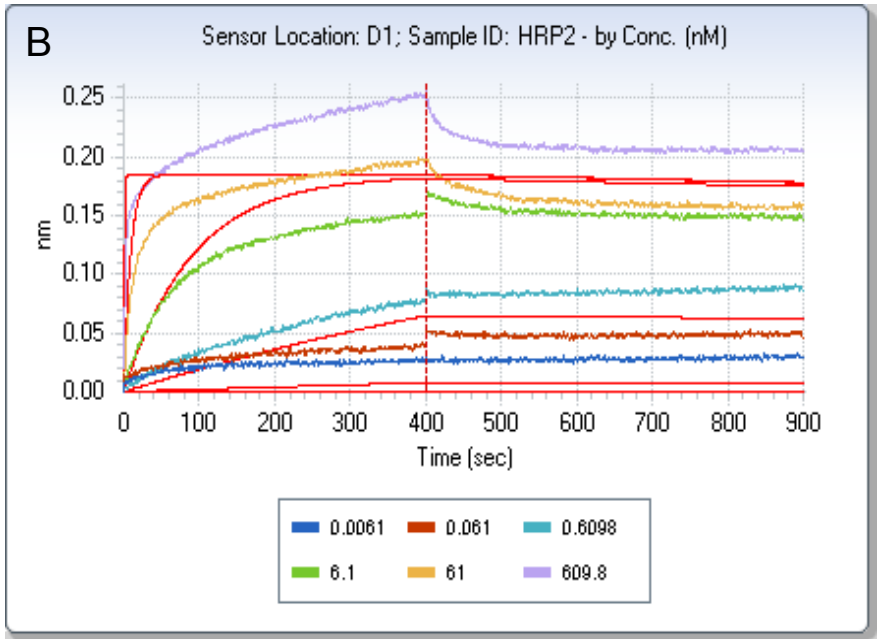

Supplement: Additional file 5 — Determination of mAbs affinity by Octet sensogram. Sensogram showing two mAbs binding to serially diluted rPfHRP2 proteins from 600 nM to 0.0061 nM. mAb D2 is represented in panel A. mAb F9 is represented in panel B. Coloured lines represent the binding interactions of mAb to different concentration of rPfHRP2. Red lines represent the statistical fitting of curves. [file 1475-2875-13-277-S5.pdf]
